# Improving adult behavioural weight management services for diverse UK Black Caribbean and Black African ethnic groups: a qualitative study of insights from potential service users and service providers

**DOI:** 10.3389/fpubh.2023.1239668

**Published:** 2023-11-23

**Authors:** Maria J. Maynard, Oritseweyinmi Orighoye, Tanefa Apekey, Ellouise Simpson, Margie van Dijk, Elizabeth Atherton, Jamie Blackshaw, Louisa Ells

**Affiliations:** ^1^School of Health, Leeds Beckett University, Leeds, United Kingdom; ^2^Obesity Institute, Leeds Beckett University, Leeds, United Kingdom; ^3^Bradford Institute for Health Research, University of Bradford, Bradford, United Kingdom; ^4^School of Health and Related Research, University of Sheffield, Sheffield, United Kingdom; ^5^Office for Health Improvement and Disparities, Department of Health and Social Care, London, United Kingdom

**Keywords:** ethnicity, obesity, adult, health services need and demand, qualitative research

## Abstract

**Background:**

A significantly higher proportion of UK Black ethnic adults live with overweight or obesity, compared to their White British counterparts. The role of obesity in excess infection rates and mortality from COVID-19 has increased the need to understand if weight management interventions are appropriate and effective for Black ethnic groups. There is a paucity of existing research on weight management services in Black populations, and whether anticipated or experienced institutional and interpersonal racism in the healthcare and more widely affects engagement in these services. Understanding the lived experience of target populations and views of service providers delivering programmes is essential for timely service improvement.

**Methods:**

A qualitative study using semi-structured interviews was conducted in June–October 2021 among 18 Black African and Black Caribbean men and women interested in losing weight and 10 weight management service providers.

**Results:**

The results highlighted a positive view of life in the United Kingdom (UK), whether born in the UK or born abroad, but one which was marred by racism. Weight gain was attributed by participants to unhealthy behaviours and the environment, with improving appearance and preventing ill health key motivators for weight loss. Participants relied on self-help to address their overweight, with the role of primary care in weight management contested as a source of support. Anticipated or previously experienced racism in the health care system and more widely, accounted for some of the lack of engagement with services. Participants and service providers agreed on the lack of relevance of existing services to Black populations, including limited culturally tailored resources. Community based, ethnically matched, and flexibly delivered weight management services were suggested as ideal, and could form the basis of a set of recommendations for research and practice.

**Conclusion:**

Cultural tailoring of existing services and new programmes, and cultural competency training are needed. These actions are required within systemic changes, such as interventions to address discrimination. Our qualitative insights form the basis for advancing further work and research to improve existing services to address the weight-related inequality faced by UK Black ethnic groups.

## Introduction

Data for England suggests that 73.6% of Black ethnic adults live with overweight or obesity, compared to 63.3% of their White British counterparts, with highest prevalence among Black Caribbean and Black African women (1). Evidence indicates that the disproportionately high COVID-19 infection, morbidity, and mortality rates faced by some racially minoritised groups in the United Kingdom (UK) and elsewhere (2), may in part be explained by higher prevalence of overweight, obesity and associated comorbidities (3–6). Analysis of the UK Biobank study suggests that at a body mass index (BMI) of 35 kg/m^2^ [BMI exceeding 30 kg/m^2^ indicates obesity (7)], the odds of contracting COVID-19 were 2.56 times higher for minority ethnic individuals compared with White Europeans (8). An increased BMI was more strongly associated with mortality among Black Caribbeans compared with White British groups (9). Associations cannot determine whether obesity is an independent risk factor for adverse COVID-19 outcomes, but plausible mechanisms linking excess fat to more severe COVID-19 infection include altered immune response underlying cardiovascular, respiratory, and metabolic disease, such as type 2 diabetes (T2D) (9). Globally, the rise in T2D in the last four decades has mirrored the steep rise in obesity (10). Overweight and obesity are therefore acknowledged as significant risk factors for T2D and associated complications (11). T2D risk is estimated to be 2–3 three times higher among populations originating from sub-Saharan Africa living in Europe, compared to White ethnic groups in host countries (12). Further, a UK study has compared the incidence of T2D found at a BMI of 25 kg/m^2^ in White populations with cutoffs for other ethnic groups [BMI exceeding 25 kg/m^2^ is the conventional cutoff indicating overweight (7)]. In Black populations, for the equivalent age-adjusted and sex-adjusted incidence, the BMI cutoff was 23·4 kg/m^2^ (13).

Mainstream behavioural or “lifestyle” weight management services available for adults living with overweight or obesity in the UK, known as Tier 2 services, are multi-component diet, physical activity and behavioural change programmes. These services are usually commissioned by local authorities, and by the National Health Service (NHS) for patients with co-morbidities, but there are also locally available services that participants pay for out of pocket. However, there is no published evidence on whether these services resonate with and are effective among ethnic groups including Black African and Black Caribbean populations. This reflects longstanding calls for tailored health programmes for underserved ethnic groups to tackle modifiable risk factors in the context of existing socio-economic inequalities (14, 15) and that weight management research should aim to identify differences in effectiveness of programmes, based on socioeconomic status, age, gender and ethnicity (16). There are local, tailored services being delivered (e.g., UP!UP! in London: https://councilmeetings.lewisham.gov.uk/documents/s105437/Appendix%201%20UpUp%20Weight%20Management.pdf); however, they are new and not fully evaluated, or otherwise are not within the formal evidence base.

To the authors’ knowledge there are no existing, widely implemented, weight management programmes for UK Black populations. One lifestyle management programme Black Africans and Black Caribbeans living with T2D has been identified (17). The Healthy Eating & Active Lifestyles for Diabetes in African and Caribbean communities (HEAL-D) study has demonstrated that it is feasible to recruit and randomise the target groups into a culturally tailored diabetes self-management programme (17). A multicentre, randomised controlled trial of the programme is currently underway.^1^ HEAL-D was developed through participatory co-design which included the use of focus groups and interviews (18). These discussions indicated, for example, the importance of nurturing collectivism, preference for verbal and visual information, and avoidance of technical or medical terminology in the content and delivery of the intervention (18). Such formative work demonstrates the value of garnering stake holder insights, particularly in qualitative research which is limited among minoritised ethnic groups. Potential service users’ perspectives from the small number of relevant qualitative studies included appearance as a motivator for weight loss (19, 20); walking, dancing, and small portion size used as weight loss approaches (19, 21); the need to address time constraints and cultural barriers to services (19–21). These findings in relation to weight management align with reviews of barriers and facilitators of diet and physical activity behaviour change among minoritised ethnic groups, including Black ethnic groups (22–24). Providers’ viewpoints in previous research have indicated the need for lifestyle management interventions for Black British populations to become embedded within current primary care structures and to align with healthcare professionals’ incentivised targets/ metrics (25). Although highly valuable contributions, a focus on weight management among the general population of Black ethnic groups living with overweight and obesity is a significant gap in the existing evidence base.

There is an overemphasis in the literature on genetics, cultural practices or beliefs as explanations of ethnic differences in health outcomes that have not been rigorously tested (26, 27). Such patterning, however, is shaped by wider issues of disadvantage and intersect with other characteristics such as age, generational status, gender/ gender roles, migration status, and experiences of racism and other discrimination (26). The central importance of structural, institutional, and interpersonal racism is often overlooked when considering the health of ethnically diverse populations (27, 28). The murder of George Floyd in the USA in 2020, and the disproportionate impact of COVID-19 on racialised minorities, has exposed longstanding systemic discrimination in ways which are difficult to ignore (29). The responding resurgence of the global Black Lives Matter protests are a stark reminder that not only does racism remain a present factor in health inequalities, but that the voices of those impacted need to be heard (30).

Thus, an overarching anti-racism and social justice lens (31) was applied to the current study. This approach incorporates an understanding of how structural, institutional, and interpersonal racism shapes everyday reality, and calls for restorative action to realise equitable resources, fairness, and eradication of discrimination. The overall aim of this qualitative study was to understand the lived experience of Black African and Black Caribbean populations, and the views of weight management service providers, to inform service improvement for these target groups. Specific objectives were to (1) Understand the experiences of Black ethnic adults living with excess weight, gaining insights into key drivers of weight gain and support needs; the enablers of and barriers to access, and engagement with, both privately and publicly funded weight management services; and what ideal weight management support looks like to the target populations; (2) Explore the views of weight management service providers to further elucidate the cultural competencies, communication and delivery mechanisms required to support effective programme development and implementation among Black ethnic adults; and (3) make recommendations, shaped by participants’ voices, for improving equity in weight management support for Black women and men.

## Methods

### Study design

A cross sectional qualitative research design was used. Qualitative approaches aid understanding of the meanings people attach to actions, decisions, beliefs and values within their social worlds (32). Gaining that understanding, and increased public involvement in decision-making, are recognised as essential in the design and delivery of person-centred health programmes and services (33). There is no one type of qualitative research. The approach used depends on a number of factors including researchers’ views on what can be known about the social world and how that knowledge can be acquired (32). The perspective we have taken is that, within an anti-racist and social justice framework (31), it is acknowledged that ethnic health inequalities are shaped by wider socioeconomic and other disadvantage which in turn intersect with personal characteristics, practices and beliefs (34). As such, the study is underpinned by a critical realist stance encompassing both objective realities and the need to centre the subjective experiences and perceptions of underserved populations (35). Data were collected in one-to- one interviews from June to October 2021 to address the research aim and objectives. Due to ongoing COVID-19 pandemic restrictions at that time, interviews were conducted via video conferencing or phone. Reporting of the study was guided by the consolidated criteria for reporting qualitative studies (COREQ) 32-item checklist [(36); Supplementary File 1]. Ethical approval for the study was obtained from the Research Ethics Committee, School of Health, Leeds Beckett University (Ref number: 85617).

### Setting, sampling, and recruitment

As interviews were conducted by remote methods, anyone meeting the eligibility criteria in any UK location was able to participate. Two samples (one of potential service users and the other of individuals in weight management service provider roles) were required to address the objectives.

Authors OO (the study research assistant and doctoral student) and TA (a post-doctoral researcher with over 10 yrs. experience), both of Black African ethnicity, MJM (a post-doctoral researcher with over 20 yrs. experience and the research lead) and ES (a graduate senior dietitian and member of the Migrant Health Public Involvement in Research (PIR) group), both Black Caribbean, all female, utilised their links with faith and other community organisations, and professional connections more widely to recruit both samples. Awareness of the study was raised through sharing a study poster via email and social media. Some respondents were previously known to the researchers, but no discussion of research goals, beyond what was in the study materials and clarifying any queries, was entered into prior to the interviews and the onus was on potential respondents to register interest in taking part.

A purposive sampling approach was used from June to September 2021 to obtain the potential service user sample (hereafter the “participant” sample), stratified by ethnic group (Black Caribbean or Black African), age and gender, with the aim to iteratively recruit approximately 12–20 individuals. Adults over 18 years and with a desire to be a healthier weight through losing weight were eligible. A short questionnaire (see below) was used prior to interview to obtain self-reported BMI and/ or waist circumference which puts them at risk of obesity-related ill-health according to revised cut-points for Black and South Asian ethnic groups (≥23 kg/m^2^; men= ≥90 cm; women= ≥80 cm); respectively (37) to check that we were not encouraging anyone to participate whose weight status was normal or underweight. Men and women, regardless of whether they were current weight management service users or not (e.g., former users, those using self-management weight loss aids, or contemplating using services or self-management aids), were appropriate study participants. Contact details of the study team were provided for potential participants to register interest. Similarly, the sample of individuals in weight management service provider roles (hereafter the “service provider” sample) were iteratively recruited, stratified by those who do and do not have experience of a tailored approach to the inclusion of Black African and Caribbean populations eligible to participate. Sampling continued throughout July and August 2021 until a sample of approximately 6–10 individuals was achieved. We focused on service providers in the Leeds area (where the academic team are located) but, as with the participant sample, use of video conferencing data collection methods facilitated inclusion irrespective of geographical location. The overall target sample size was chosen to provide diverse views on the phenomena of interest (38), as well as likely to be sufficient to reach data saturation (39). Due to the use of convenience sampling, it is not possible to know how many potential respondents were aware of the study and therefore the response rate cannot be estimated.

### Data collection tools and procedures

Participants and service providers completed a brief questionnaire with socio-demographic items (age, gender, ethnicity (own or, if born in the UK, parental place of birth, to further elaborate ethnicity), education, employment status/ job role, location, and, additionally, length of time in role for service providers), self-reported height and weight (from which BMI was calculated), and waist circumference. Questionnaires were provided via a Google Form, but hard copies and support from researchers to complete the questionnaire were available if preferred.

A semi-structured guide for the participant interviews was developed based on qualitative evidence relating to behavioural weight management among adults from minoritised ethnic groups in the UK (19–21). These studies aided our understanding of topics to cover and areas to probe, for example motivations for behaviour change (19), barriers and facilitators to healthy lifestyle (21), and attitudes towards weight and weight control (20). We also drew on other literature on subjects ranging from access to healthcare to beliefs about racism and health (40–44), and consultations with the Migrant Health Research Patient and Public Involvement (PPI) group. Topics included life history and migration narratives; racism and other discrimination; social constructions of health/ healthy weight; exploring associations between life stories, health, and weight; motivation for weight loss; awareness of and access to weight management services; barriers and facilitators of weight loss and related-behaviours (addressing objective 1); and views on ideal services (objective 3). Similarly, published studies were used to develop a broad topic guide for the service provider interviews. These included studies that targeted low socio-economic populations more generally, lifestyle programmes other than weight loss (such as T2D support), or were conducted in settings outside Europe (25, 45–47) Topics included the nature of the weight management programme and their role; programme participants; recruitment strategies; cultural tailoring/ adaptation; cultural competency; programme effectiveness; barriers and facilitators of access and effectiveness; recommendations for addressing barriers (objectives 2 and 3). The guide was further developed with the incorporation of vignettes [short descriptions of people, events or circumstances provided by the researcher to prompt discussion (48)] from participant interviews and questions directly relating to the vignette content. Both interview guides (Supplementary File 2) were discussed by the research team after the first two interviews in each sample, and it was deemed that no changes were required for the main data collection.

Prospective participants who registered interest were sent a participant information sheet, consent form and the questionnaire. Data collection proceeded once informed consent was certified with a completed, signed and returned consent form. Eighteen participants and 10 service provider interviews were conducted by OO, ES (previously trained by MJM), and MJM. Interviews were conducted in English; the team can also accommodate Yoruba, broken/ “pigeon” English, and Caribbean patois, however the rare occurrences of these forms of speech were generally during informal conversation before the interview commenced. Interviews took place on Microsoft Teams or Skype 4 Business video-conferencing platforms, or by phone as preferred by respondents. Field notes were taken to aid the analysis process, and interviews were recorded. Discussion length ranged from 24 to 66 min.

### Data management and analysis

Interview recordings were transcribed verbatim and a blended analytical approach was taken. A critical realist approach (reporting an assumed reality evident in the data) was used with inductive thematic analysis (coding and theme development are directed by the data) to identify themes and reveal deeper meaning within the data (49). The following six stages of thematic analysis proposed by Braun and Clarke (50) were employed: (1) Familiarisation with the data; (2) Generating initial codes; (3) Searching for themes; (4) Reviewing themes in relation to the coded extracts (Level 1) and the entire data set (Level 2), generating a thematic “map” or coding tree of the analysis (see Supplementary File 3); (5) Defining and naming themes, refining the specifics of each theme, and the overall story the analysis tells, generating clear definitions and names for each theme; and (6) Producing the report. MJM and OO coded the data independently, but augmented each other’s analysis rather than label any differences as right or wrong (49). After hand coding the data, a framework approach was used to chart the coded extracts to aid checking for data saturation (51). Data collection ceased when no new themes were identified in the data and saturation was deemed to have occurred. Due to the timeframe of the study, respondent validation of the findings was not conducted.

## Results

### Sample description

Eighteen participants (14 women and four men) and 10 service providers (all women) took part in the study (Table 1). Participants included 11 Black Africans and 7 Black Caribbeans, ranging in age from 24 to 71 years. The service providers, of whom half were of White ethnicity, were from a range of professions including dietetics, health care/ nursing, and health coaching, in private, public sector (internally delivered and externally commissioned), weight management services, at various levels of seniority, and with length of experience ranging from 6 months to 10 years. Seven of the service providers worked with diverse ethnic groups, with four experienced in culturally tailored approaches. The majority of the sample were born in the UK and lived or worked in English (Birmingham, London, Leeds, Manchester), or Scottish (Glasgow) cities.

**Table 1:** Sample description.

	Participants (*n* = 18)	Service providers (*n* = 10)
***Born UK, n (%)***	10 (63)	8 (80)
***Ethnicity (own/parent place of birth), n***		
Black African/ Black British (Nigeria)	7	1
Black African (Other African^a^)	4	0
Black Caribbean/ Black British (Jamaica)	3	2
Black Caribbean (Other Caribbean^b^)	4	1
South Asian (Pakistan)	N/A	1
White UK	N/A	5
***Gender, n* (%)**		
Women	14 (78)	10 (100)
Men	4 (22)	-
***Age, range years***	24–71	24–58
***Body Mass Index, kg/m*** ^***2***^ ***, median (range)***	28.6 (23.6–38.8)	27.00 (19.7–34.3)
***Employment status, n (%)***		
Works fulltime (including self-employed)	9 (50)	6 (60)
Works part-time/ as a locum	–	4 (40)
Unemployed	5 (28)	–
Retired	1 (5)	–
Full time student	3 (17)	–
***Highest level of education, n (%)***		
University (under-or post-graduate)	14 (78)	10 (100)
School level only	4 (22)	
***Number of years as a service provider, median (range), years***	N/A	4 (0.5–10)
***Practitioner role, n***	N/A	
Dietitian/ Specialist diabetes dietitian		4
Health coach/ Senior health coach		3
Community nurse		1
Health promotion manager		1
Healthcare assistant		1
Experience of tailoring services for Black ethnic groups		4

a Uganda, Mauritius, and Ghana.

b St Kitts & Nevis and Dominica.

Reporting of the themes is supported by direct quotes from the data, labelled with a pseudonym, age range (participants) and role (service providers). Diversity of views and experience is captured within the themes (e.g., different experiences of providers from Black vs. White ethnic groups). However, there were few striking differences in perspectives between participant subgroups (i.e., African vs. Caribbean; UK-vs. overseas-born). The five main themes identified in the analysis were as follows:

1. Positive about life, but in the shadow of racism
2. Weight gain attributed to unhealthy behaviours and the environment
3. Acceptance of larger body size in the context of complex weight loss motivation
4. Self-help preferred to formal weight management programmes to lose weight
5. Ideal weight management support is community-based, culturally tailored, and flexible

#### Theme 1. Positive about life, but in the shadow of racism

Those born abroad expounded on difficulties, such as the *“culture shock” (Mirembe, 35–39 yrs.)* experienced on migrating to the UK. Despite this, living in the UK (whether born abroad or born in the UK) was generally a positive experience for participants, with education and work opportunities as key motivators for their parents’ or their own migration,

“Yeah, coming to the UK as a student again…it was quite…it was good. I was just glad to be back here. For me, it is a breath of fresh air, to be honest, so yeah. And here I am, so blessed with a good job now, I’m just thankful for that. Yeah. That’s Nice. [Laughs] Yeah. So, it been a long journey, but eventually I still got to where I envisaged I would get through after a lot of sacrifices here and there” (Ese, 50–54 yrs.).

However, the reality of UK migration also included downward social mobility for some, relating to qualifications from home countries not being accepted or given consideration and other discrimination. UK and overseas born participants’ exposure to racism in various forms, including blatant interpersonal abuse, more subtle micro-aggressions, and structural racism, was related in vivid detail,

“Well, we moved to live into [an area of Leeds], then we had our mortgage. So, where we have a mortgage now, the White guy will come to us, that this is not for Blacks it’s for Whites, that we need to move, that they do not want us here. They will go and buy sweet-and- sour sauce and splash it on my door. They would throw the stubs of their cigarette without quenching the fire, they would post it through my letter box because they want the house to burn because it’s Blacks that live there. In fact, there are some White guys, my immediate neighbour was a White guy, but he moved away because he does not want to stay near Blacks. They’ll bang on your door – before you go out, they will run away” (Yetunde, 55–59 yrs.).

Some participants, although not negating the existence of racism *(“Oh, yes, lots of racism, discrimination, even present day, does not stop. It’s never ending” (Claudette, 50–54 yrs.))*, were clear that it did not affect their lives *(“I’m not going to let it define who I am and my life.” (Claudette))*, signalling overt or covert coping strategies as forms of resistance. However, for others, past and present racist discrimination, and worry about possible events in the future were a significant, and particular form of stress. Service providers were asked to respond to the vignette based on Yetunde’s experiences.

##### Bringing your “big self” to work

Responses varied from shock (*“My God are these true? God…”* (*Susan, senior health coach*)) at the nature of these types of experiences, to relating their own experiences of witnessing microaggressions, and other discrimination, in the workplace, for example. Some reflected on how such awareness could enhance their practice, particularly around supporting clients to address stress as part of their weight management journey. Views of service providers from Black ethnic groups showed how being themselves, sharing their lived experiences, and drawing out their clients’ experiences, such as racism and its impact, was hindered when working in the public healthcare system. This contrasted, with their experience within voluntary community work or private practice with Black clients,

“Because you are bringing the professional element. So, basically what I’m talking about actually…I’m at [names public sector healthcare employer] and at the end of it you need to bring your ‘big self’ to work, ok? And I suffer that pain, you know. You want me to bring Cherida to work? In my career so far, there’s only ever been one of me… one Black woman. Yes, there’s only been one Black woman in the team so me bringing all of me to work makes me feel slightly uncomfortable because I know they…everyone I work with is not going to have a clue. And that has an impact on how you communicate with your service users or your clients, or your patients, whatever. So, you are trying to find that balance of what the [employer] deems to be professional and what’s overstepping the mark. Now, if we go back to the work that I’m doing on my own, voluntary in my spare time, because I do not have those barriers and I know that to support my people, I’d bring Cherida…I’d bring Cherida there. And I know that by doing that, that would yield better results” (Cherida, specialist dietitian).

“Why have I been successful? It’s because I’m not shy about where I’m from. And if anything, I’ve kind of put that as, not quite made it my brand, but I’ve made it quite as…it’s very important to me to be…to embrace where I’m from” (Sabryna, private sector dietitian).

Negative experiences of racism and discrimination in the health care system and more widely also formed the backdrop to participants’ perceptions of weight management support, connecting racism with the engagement with services in Theme 4, below. Prior to the presentation of that analysis, views on weight gain and acceptance of larger body size (Themes 2 and 3) are explored.

#### Theme 2. Weight gain attributed to unhealthy behaviours and the environment

Discussions around living with excess weight were largely expressed in the context of being healthy overall, although there were reports of chronic ill health. Participants described changes in their weight over the life course, and shared their views on the causes of their excess weight. Common portrayals of childhood weight were of being a *“skinny child” (Ola, 18–24 yrs.)* and being slim until early adulthood, or less commonly adolescence. Older participants considered overweight to be an issue occurring in later life. Hormonal changes and the slowing of metabolism with age were considered to make weight gain harder to address compared to earlier years when weight would *“drop off”* naturally, regardless of whether it was thought necessary at the time. However, physical inactivity and less commonly in conjunction with overeating, or combined with hormonal/ metabolism factors, was the firm view of participants as the cause of their overweight,

“Lack of exercise, okay! That is my major thing, it is lack of exercise, I sit a lot. I sleep a lot. And I eat a lot of sugary things. Yes, because I do not eat normally, healthy food normally all the time” (Mirembe, 35–39 yrs.).

Family history or genetics as the reason for obesity was a view put forward by only one participant. By contrast, the main causes of overweight identified by participants were modifiable factors. Despite BMIs over 30 kg/m^2^ being common, weight was generally viewed by participants as a recent concern, with reference to lifelong weight challenges uncommon. Feeling an urgent need to make progress with weight loss and gain control was a common prompt for joining the study. Explanatory models of weight gain also centred around life in the UK compared to life in home countries for those participants who were born overseas. Predominantly, it was felt that life in home countries was more active with lack of easy access to convenience/ fast foods, more consumption of organically grown food and less consumption of processed food. Additionally, participants suggested that a less stressful lifestyle in home countries contributed to not having weight problems until migration. There were contrasting views among some Nigerian participants that life was *less active* in Nigeria than in the UK, indicating that home country urban/ rural and social class differences impacted on experiences, as these quotes suggest,

“I think that in my country of birth, I was healthier because I did not eat a lot of processed food, it was cooked, hmm, natural, somehow natural foods. I…we did not eat a lot of packaged or processed foods, it was food that was grown and cooked. And also, I walked more, and I was more active in my home country” (Ola, 18–24 yrs.).

“I think in Nigeria, we do not exercise. I cannot remember in Nigeria ever exercising, if you know what I mean, and I was driving, so if I needed to go to the supermarket, which is probably compared to the UK, I’d probably walk. Nigeria, I just drive down. And I was always driving everywhere, you know. So, there was no opportunity to walk. I tried of course, we had, you know, access to clubs and all that where you could go swimming, do sports. But then one never really had that time. And it is maybe you want to just give the children a treat, you know, it’s OK. We are going to the club today and we are going to, you know, swim. We will go and eat something nice; you know and all that. But outside of that, I think in Nigeria, exercise is a big problem” (Ese, 50–54 yrs.).

The discussion further elicited views about relationships with food and interest in physical activity, more generally. Prompting detailed descriptions of dietary habits was not a study aim, nevertheless, a love of food and cooking was expressed across the sample, with most claiming a healthy relationship with food. However, emotional eating in relation to stress or “lockdown” related boredom was also mentioned. Commonplace barriers to healthy eating were the perceived cost of healthier food choices and the easy availability of fast food. *“Mindfulness”* and *“conscious eating”* were mentioned as aiding healthy choices. Traditional eating emerged as occupying contrasting moral spaces [i.e., having both positive and negative moral significance (52)]. Traditional foods, which represented sense of self, community, and links with “home”, were highly valued. The potential for being deprived of, or sacrificing these foods, was at the crux of dissatisfaction with weight management advice and resources. However, for some participants traditional dishes, and starchy foods in particular, were newly framed as villains needing to be reduced or removed within self-help approaches to weight loss. This caused the potential for inter-and intra-personal conflict, such as described by this gentleman,

“Yesterday I prepared egusi soup [stew made from melon seed, oil and spices to which meat is added] and yet I am so scared of going to eat eba [cassava accompaniment], because of my weight” (Ubong, 65–74 yrs.).

Discourse around physical activity ranged from reports of lack of interest, or limited engagement with available facilities, but more usually involved the description of a range of activities that participants liked to do. Preferred activities included cycling, swimming, and dance-based exercise classes, with walking the most favoured activity. Walking as part of the commute to university or work was cited as an incidental activity contributing to weight loss goals. Aside from walkable commutes, there was limited discussion that could be credited as facilitators of physical activity, but this included advice from a friend or being a participant on another research project. Barriers to living a more active life included cost (e.g., of gym membership or swimming sessions), stress (e.g., work-and or family-related), lack of time, lack of knowledge about or existence of facilities, and fatigue.

#### Theme 3. Acceptance of larger body size in the context of complex weight loss motivation

Across the diverse cultures and place of birth within the sample a positive view of larger body size among themselves and family members, was commonly referenced,

“I think my father had a pot tummy, but I did not consider my mother fat. I think that her tummy was big and I think it was a normal thing, you know, what they call evidence of good living” (Amma, 55–59 yrs.).

“As I said, that’s my family, they are predominantly big – fat people you know – if I go too light in weight, I look like I need to fatten up, as my mom would say [laughing] You know West Indians…” (Claudette, 50–54 yrs.).

These ideas around body size were held alongside, not instead of, motivation for weight loss. This included health-related motivations, principally preventing stroke and T2D, as well as maintaining mobility. Family history of overweight was emphasised as an impetus to lose weight, rather than as a cause of their excess weight, as noted above. However, the principal motivation for weight loss (i.e., mentioned first and with emphasis) was being able to fit into a smaller dress/ shirt size, to improve appearance, feel comfortable and look younger. This apparent contradiction may signal a tipping point at which the “body positive” view loses cogency, and/ or health takes on greater salience. Further, for some these seemingly conflicting or contrasting views could be an indication that a positive view of large body size may be public discourse that conceals more privately held body image dissatisfaction, converging with an outlook more commonly associated with Western cultures,

“I’ve always been thin [when younger]. So, most times…when I came here…they used to say I was underweight, but it’s about two, three years, I’m not happy with my weight, I see it’s coming on. They used to say I am underweight, but I used to love it that way [laughs] I am gaining, I hate it so much. […] I hate my weight. I hate it, I want to be slim, I want to see myself to wear some clothes and I’m like, Yeah, I’m spot on, you know…We shop online, and you see the models. Yeah, they are wearing, what you want and you are like, Yeah, I want that! And even you have to try to look like that model. So that’s my main thing to look nice and young [laughs]” (Mirembe, 35–39 yrs.).

##### Preference for alternatives to BMI measures

Linked with views on body size, there was matter of fact acceptance of the concept of BMI by some. However, more often there was criticism and the view that BMI is a threat to positive body image. It was also considered a professional/ medical concept that differed from lay perspectives, and is *“too stringent”* (*Oluseyi, 35–39 yrs.*) for Black people,

“Essentially, I do not, personally I do not see the relevance for Black people in the way that we are built so I use the waist: hip ratio, this kind of measure because I feel like even my brother who’s quite healthy, he goes… because he’s quite muscly and is quite big, he’s obese [according to BMI] but he does not look obese, if that makes sense? So, I do not, I do not, I just kind of ignore this stuff, I’m not going to lie to you, yes” (Chinwendu, 30–34 yrs.).

On the whole, the service providers were sympathetic to this view and some also preferred measures such as waist: hip ratio as BMI alternatives.

#### Theme 4. Self-help preferred to formal weight management programmes to lose weight

Self-help approaches to weight loss were common, particularly among those who considered weight loss to be a personal issue or did not conceive living with excess weight to be a health problem in itself. Consistent with views on causes of excess weight, approaches to weight loss involved cutting down on food consumed and increasing activity. Methods included, intermittent fasting, cutting down on carbohydrates including eating less traditional foods (as noted above), using meal replacement shakes, alternative remedies, and supplements, as well as monitoring techniques such as daily weighing. Digital content such as YouTube videos and advice posted online by service providers in home countries was relied on for support, partly prompted by lack of cultural tailoring within UK services (see *Lack of tailoring and relevant resources* subtheme, below). Little was proffered in the dialogue related to this online advice about what was considered credible and what was not,

“I feel like things like nutrition so, for example, like when I was trying to lose weight and stuff, it can sound so rude but, the menu was very much prominently for White people, so, they would do something like soup or baked potato and tuna, do you get what I’m saying? Now, currently, what I do is that I’m on a diet from this lady in Nigeria so, it’s been easier for me to stick to it because it’s food that I eat and then after, like, because when you are talking to the GP and stuff about African food, they just assume ok, yes, the food has lots of oil and, do you understand what I’m saying? So, I feel like this, just to cut it out to me but they do not look, they do not really study the food, I do not know if that makes sense? [Researcher: Yes]. Because not all African food is bad, I mean, take Jollof rice, for example something like a bowl of rice and tomato sauce, because it’s rice, you just have to eat it in moderation. Yes, that’s what I would say in regard to that, I think that when it comes to them giving advice about health and all of that stuff, they kind of just use the food that’s available in the UK and I do not think they take into consideration your culture” (Chinwendu, 30–34 yrs.).

##### Poor communication of weight management services

The most significant barriers cited by participants to accessing and engaging with weight management services was not knowing about the existence of programmes, and a lack of close, personal support. Service providers also acknowledged limited communication about the availability of services,

“They do have self-referrals as well…a person can, if they feel they need it, but what I do find is (1) nobody knows about that and (2) they do not know where to go. If they have got a friend or family who works at the NHS, maybe they do but the general person, I find they do not know that they can self-refer” (Cherida, specialist dietitian).

Where there was awareness of formal support there was some distrust of self-funded commercial services and the view that they were a waste of money, out of reach financially, or both. Few participants were aware of publicly funded services. Any naming or descriptions of the content of services that are freely available were largely hesitant or vague,

“So [names a weight management service provider commissioned by NHS] is a group that helps with, eh, what do they do now? They, they, help you manage to live healthy, if you like, where you can record what you are eating, your weight […] and you’ll have a coach supporting you” (Tosun, 40–44 yrs.).

“Umm…I know it’s in the community because they have exercise classes and different sort of stuff through my neighbourhood scheme. OK, I know at the hospital they do offer wellness, support and classes, yoga, and stuff like that if you wanted it as well. And it’s free. So I am aware that with that they are there” (Claudette, 50–54 yrs.).

Other than being freely available, participants indicated that there was little external incentive to engage with public sector services. By contrast, service providers in private practice described the various ways in which they used freelance directories, social media, word of mouth, and methods to boost website traffic to increase awareness of their services together with incentives to boost their client base,

“Yeah, so incentives. So definitely social media, I have done kind of like discount codes in the past. And just kind of have those, around like independence days. So, like, kind of like, it might be St. Lucia’s Independence Day, and we are all you know, is I cannot remember the ad now, but it was actually quite a lot of interest in it definitely resonated a lot more with an older population as well within that African-Caribbean group. Because I guess that ‘hashtag’ at that moment in time, you know, was quite popular. So then my ad went with that. Yeah. That was, that was quite good. Yeah, so that’s been and then that would have a discount code attached to it. But there’s other incentives I could do. It could be aligning with other, like, Afro Caribbean companies or restaurants and offering via discounted food with a consultation and I would just think things around that” (Sabryna, private sector dietitian).

Few of the participants had attended formal weight management services, and among those that had descriptions of their experiences did not indicate any deep commitment to attending these types of programme. *Yetunde*, who also previously engaged with an exercise referral scheme, described a positive experience of a weight management service, particularly around the psycho-social support embedded in the programme,

“Unfortunately for me I do not know, you know, then because of the stress that I was having, I lost interest in doing this [exercise]. I used to be very sporty, but because of stress, I was not doing anything, I put on weight, I was not doing any exercise. Then fortunately, my GP was able to pick it up, when they did an annual health check for me, they discovered that my blood sugar was going up. The greatest thing my GP did for me was to introduce me to [names provider], the NHS [−backed] weight management programme. That [names provider] taught us a lot of things. That was where I discovered the problem, I had then was due to stress. You know, I was doing emotional eating, I was doing emotional shopping. You know, just to comfort myself, you know, all this kind of thing” (Yetunde, 55–59 yrs.).

As was the case for Yetunde, the role of GP consultations was a common topic among participants’ engagement perspectives, alongside GP referral among service providers. Primary care had a contested position among participants as a source of weight management support.

##### Missed opportunities in GP consultations and referrals

GP practices were considered the first port of call by some participants for weight related issues, but there was disappointment in the level of advice or support given. Others were adamant that the GP would not be appropriate. There was a perception among some participants that they received good treatment from their GP because they did not live in *“working class Black areas” (Oluseyi, 35–39 yrs.)* where they felt overt racism was more likely. In keeping with that view, for others racism was a barrier to getting good service at the GP practice, with participants linking this to living in an ethnically diverse community,

“Yes. I think based on some of the experiences we have had with our practice, GP practice, I think it’s there because of the area where we live. We are in a GP, where we live, which is it is highly populated by, you know having the BAME population. I think the care is not as good as the areas where you may have more Caucasians, Whites. So, the kind of attention or the kind of response you get even when you call, is I feel it’s also biased and probably due to that. And so, again, that is it. How do I put it, that, that’s a barrier to some kind of access and because I would think twice about calling them, if you know what I mean?” (Ese, 50–54 yrs.).

Service providers working in the public sector explicated the challenges associated with the GP referral process. These included underdeveloped relationships between general practice and weight management service providers, a focus on co-morbidities, particularly diabetes and non-diabetic hyperglycaemia (“prediabetes”), as driving factors for referral, long waiting times between referral and starting a programme, and clients not sharing their GPs view of the need for weight loss. Such challenges, as illustrated below, potentially contribute to missed opportunities for primary care referral to foster weight management service engagement,

“So, it’s having that rapport with the nurses – we build a rapport with the nurses, that’s what I kind of am doing now, building the rapport with the nurses and the practice managers and we’ll just have, I’d say, basic referral criteria, certain weight or HbA1c, bloods and things like that. Previous services I’ve worked with are very much the same, how can I say? It is a bit of a tick box. Do you sit in this box, or do you sit in that box? But then, the person has to be assessed to sit in that box. So, I do find that there’s a lot of people who do not fit in any box because maybe they have not seen their GP for a while so, you know, they have not been assessed or they have gone to the GP for a cough, a cold or something else, but their weight is not the main purpose of that conversation so, that [overweight] does not get looked into. So again, it’s looking at that, how are we going to adjust the criteria” (Cherida, specialist dietitian).

##### Lack of tailoring and relevant resources

The common thread across these different levels of awareness and engagement was the view that the services were not sufficiently tailored for Black ethnic groups. Views were commonly based on assumptions, or from experience of trying a programme as *Emelie* and *Tosun* indicate, respectively,

“I think to be honest you know you said that like for people that are Black, I do not think there’s any distinction. And to be honest, the way that I read about weight loss in general, it’s like everyone – this is gonna sound weird – but it’s like if you are fat or overweight, everyone’s just lumped together in this one giant ball, and then that’s it. So it’s like we are just like one homogeneous group. So I do not think in some ways…but that’s why I think to me that’s what it looks like, because at this moment in time, there is not any personalised health care, we are just not there. It’s like, I could be Black, I could be White, but I think that if you are Black, or you are White, there is a difference that you get in health care, there is a difference” (Emelie, 40–44 yrs.).

“If I had a dietary coach that is Nigerian, mind you, and then I’m bringing this portion of egusi soup, okra soup, ogbono soup [stew made from wild mango seeds with meat and fish], and she will say, ‘hey, hey, hey, this is high in this, this is high in that’ in that you cannot match these two, or you need to stop using oil for this, you need to do this, you need to do that. Then, you know, I’ll have more confidence. In all honesty, I do not have so much confidence in this programme I’m doing ‘cos, having said that, I have learned a thing or two from it, but in totality, no, I would not say I have full confidence in it, not any fault of the person, the coach per se, but it’s just the fact that the proximity in terms of food we are exposed to eat is different” (Tosun, 40–44 yrs.).

Across the board there was the sense of limited resources or advice that accommodated participants’ preferred food choices, particularly how to continue eating their traditional food. Service providers, though refuting any notion that traditional eating could not be incorporated into weight loss plans (see above), acknowledged the dearth of suitable materials, their own lack of training, and relying on clients to educate them,

“It does generally show you in the diet [plans] of White British diet and that kind of thing, you know, it does not include any of the traditional food, I think. And I think for that information, you do have to do a bit of digging, you know, and I know the information is out there but it’s not, it’s not really commonly promoted, I would say. So, you know, if they kind of see that initially, they might go on to the [name of charity] website or might go […] and see that kind of thing, and think and say well, this really is not for me, these aren’t the kinds of food I eat every day in my diet. So, you know I’m not sure this would be good fit” (Monica, health coach).

“I think that’s something that maybe we do not get enough training of as, kind of, nutritional professionals, we do not have much awareness, this might be kind of a generalisation but there’s not much awareness of the minority ethnic groups and their traditional foods and kind of what they are, it’s normally quite western in the approaches that we have. And I think the example here [referring to the vignette] is probably, yes, it’s putting me on the spot, and making me feel maybe we should be a bit more aware of world food, not just food in the western world” (Jill, health coach).

Participants and service providers working in the public sector commonly proposed working closely with communities to improve engagement. These and other ideas on what ideal weight management services should look like are explicated in the final theme.

#### Theme 5. Ideal weight management support is community-based, culturally tailored, and flexible

##### Community engagement

Working more closely with local communities was considered by both participants and service providers to be an important way of addressing the apparent lower engagement of Black ethnic adults in weight management services. Service providers described nascent plans or indicated that ideas had been mooted in their organisations, but concrete examples of this taking place could only be provided by those from Black ethnic groups doing voluntary work,

“I think we maybe could be doing more community work which is something that we are planning on” (Jacqueline, head of health promotion).

“That’s another thing, before the pandemic, I was doing workshops at churches across my free time and that went down really well, if I do say so myself. And I do appreciate that they felt comfortable just to have a general conversation with me and I really think is the fact that a lot of people go to the clinic, they do not want to come across as uneducated or not aware, you know, and they leave […]. So, that’s the project that I’m working on and hopefully, once the pandemic ends, it could go really well” (Cherida, specialist dietitian).

Participants involved in community organisations (which included faith, cultural, and health-related support, and outdoor activity groups) could see ways in which raising awareness of weight management services could be incorporated into the organisations’ activities, suggesting that some potential mechanisms or gateways for engagement are in place,

“So, ideally, I would say, oh if you know any group, for instance, the [anonymised community organisation] in the UK, what have you, if you have a good, creative forum, this is so easy. Everybody come on and then we talk about weight management and talk about health care, we’ll talk about this and that, and that would bring into people’s consciousness where they are and what they need to do. And that would save a lot of lives and you know with social programmes, again, it would bring about change of lifestyle clearly, it would save lives, that’s just the bottom line, so, my suggestion would be for people like you who are carrying on social services for instance at this particular time, it might just part of your…or you are doing this now, if you then go on to take this a step further, just say Ok, I’m going to give support to this group and then you speak with, with the group leaders and say Ok, this is what I would like to do for the group and then you create this platform where you can talk about this issues that we have here and why it is important then, yes, it would be really good, really good” (Tosun, 40–44 yrs.).

“I think maybe, you know, there could be other ways in terms of like community groups and, you know, promoting it through there. I think they could be good so if the individuals are around us, you know, if they trust, they can come by and have a conversation with us and that, you know, might have might open some doors, I think” (Monica, health coach).

Community-based communication could also potentially open up the conversation around prioritising weight related health issues and combat stigma,

“I think the only [other] thing for me, just speaking to you, that stood out to me is about the weight management concept. I do not think that that’s a concept that many people really consider. I think they see as a personal issue that they…they need to solve themselves unless the doctor themselves told them actually, you need to do something about your weight. Then I think, even if somebody was obviously obese, I think there might be some barriers so, you know, being ashamed or feeling inadequate when it might not be opened to getting that help anyway so I think the concept of weight management needs to be mentioned a bit more. I do not think it’s sort of, at least not for me and people that I think we have talked about among ourselves as friends, as women, we do not, I do not say oh, I think my friend is telling me up or I’m doing this, I’m doing that, you should go and see your GP. I do not think, I do not see it as that. So, I think it needs to be mentioned more” (Kemi, 30–34 yrs.).

In addition to community-based awareness raising, suggestions for locally placed programmes also intersected with other needs. Local services could partly address financial barriers to accessing privately or publicly funded services by avoiding the time and cost associated with travel, including the cost and inconvenience of public transport for those without car access. Local, community-based services were also posited as more likely to be inclusive and attract diverse groups (so that no-one would be the *“only Black person there”* (*Ola, 18–24 yrs.*)) and together with flexible delivery could help accessibility within complicated work, home, and wider caring responsibilities.

Within the context of community engagement, face to face delivery was commonly asserted as essential for services to be relevant and would provide opportunities for wider social engagement. Bound within that view, however, was the acknowledgement that some remote delivery was necessary, particularly within the context at the time the research was conducted of continued control of the COVID-19 pandemic. This also coincided with the need for flexibility, and for some, privacy, a preference for self-help, or both. Weight management service providers acknowledged both advantages and disadvantages of digital and remote delivery and speculated that the current focus on some forms of delivery (e.g., use of apps or video conferencing platforms) could disadvantage certain populations due to unequal access to digital technologies.

Linked with how services should ideally be delivered, the ethnicity of service providers was considered to be key and is summarised below as one aspect of cultural tailoring, together with the other essential facet which was tailored content particularly around dietary advice that is sensitive to traditional eating.

##### Cultural tailoring: ethnic matching

In terms of who delivered the services, ethnic matching was universally cited as beneficial and for some, essential, largely relating to the ability to give relevant dietary advice,

“It’s very, very important because there’s more understanding, because you are more likely to eat the food that you eat before, if that makes sense (Chinwendu, 30–34 yrs.).

“It’s better because they can relate to what you eat, you know, and probably can tweak your diet more to your needs that would suit you, you know. Yeah – I know that sounds a bit racist, but it’s not. I do not think it is because it is a matter of somebody else might not know what I eat, or what Caribbean people eat. And then, you know, you are talking of the food that they want you to eat is not what you would normally eat. You know, so I think seeing someone from your own background is better because then they can say, well, you cannot eat yam because white yam is higher, or yellow yam is lower in calories or, you know, just tweak to your specific needs. So, I think in terms of weight, you should have someone from, you know, similar to you and can relate to the foods you are talking about Yeah” (Claudette, 50–54 yrs.).

“It is quite important because when I had a personal trainer, there were certain things I was eating she could not really make sense of how I was having to explain a lot of things, it just felt like a burden. So I definitely would say it is very important” (Kemi, 30–34 yrs.).

Whereas it was acknowledged that the full ethnic diversity of the population could not feasibly be exactly matched, there were instances where participants felt matching was superficial and clumsy. For example *Tosun* (who is African) describes being assigned a Black Caribbean health coach,

“So, when I was referred to these people, they tried to say to me ‘Oh, we have people who are from Afro-Caribbean background that would understand your country food and be able to advise you’. Honestly, I do not think that has really helped. I think generally I say to myself, listen, these people would not probably understand so much of your food. For instance, if I make okra soup or banga soup [stew made with palm nut fruit, spices, to which meat or fish is added] or egusi soup, I’m trying to describe what I’m saying, or melon soup and vegetable cooked with fish meat and…My view is if you are going to make this really work for everybody, do not put a round peg in square hole. Make sure that people who are from the same ethnic background match the people who need the service. Because the best…come up with is ‘Oh, we have people from Afro-Caribbean ethnicity that can understand what you are eating’, but no, they are [from] far off – miles away from West Africa […] So, it will be better if we have that sort of close match” (Tosun, 40–44 yrs.).

*Cherida’s* and *Sabryna’s* stories (Theme 1, *Bringing your “big self” to work* subtheme, above) indicated their view on their “insider” ethnic position with regard to supporting Black people living with excess weight, and how being able to capitalise on that position might be shaped by their (public vs. private sector) work environment. Service providers of White ethnicity recognised that their own and colleagues’ identity may be a factor in not attracting Black ethnic groups to their services, but they also indicated commitment to addressing equality of access. Lack of monitoring or analysis of recruitment by ethnicity, meant that reasons for lack of engagement and success by ethnic group, and ways to address this were speculative,

“Researcher: Okay, and why do you feel that you do not have a high percentage of Black African or Caribbean clients?

Julia: I do not know. The short answer – I wish I knew the answer. I suppose possibly because I am a White woman, I’m the face of the company, largely that has not…and that can perhaps mean it does not seem like it might be seen as an inclusive space, for example” (Julia, private sector dietitian).

“I think it probably does not help that in our organisation we have very few people from minority ethnic groups, but it is something we are working on addressing.” (Jacqueline, head of health promotion).

##### Cultural tailoring: gender unimportant and weight-matching undesirable

It was clear that for participants, gender matching was regarded as less important. Although some reflected that, hypothetically, the absence of gender matching could increase self-consciousness with regard to exercise sessions, no one expressed this being a barrier for them personally. The notion of services being delivered by individuals who also live with excess weight elicited stronger views. Advice from service providers with *past* experience of living with overweight was considered potentially valuable; however, receiving advice from providers currently living with overweight was viewed as unimportant or even detrimental,

“Researcher: Ok, how important is having the health professional the same body size or shape as you?

Oluseyi: Well, I’d prefer they were not, to be honest” (Oluseyi, 35–39 yrs.).

“Well, that’s the thing, because I normally say you should practice what you preach. So if you are fat and you are talking to me about that, it’s not going to be effective for me. But then that’s me [laughs]. If you say to me, well, you were that weight and you came down to this. Oh, yes, I can absolutely relate. Then I’d say yes, definitely. But you cannot expect me to do something if you are not. No! So, OK, it’s about the public health professional understanding. ‘I know that this is the weight you are at, I’ve been there. You can get to this weight because I’ve been there and I’ve done it’, you know, but if you are fatter than me and you are talking to me about that, then it’s not going to work” (Claudette, 50–54 yrs.).

##### Cultural tailoring: programme content

Participants expressed the need for cultural tailoring of programme content, focused largely on tailored dietary advice with detailed and authentic inclusion of traditional foods and eating habits. The specific needs commonly pinpointed were an essential focus on portion size advice, tailored menu planning and modified preferred dishes to give them a more healthful energy and nutrient profile,

“Ummm, exercise programmes are general, you do not have to tweak it for anybody’s size. I think, just the meal plan and just the support, the counselling support, you know. But we as black people, we love our food. So I do not know. You’re not going to deprogram us from not loving our food. [Laughs] I think we have a love affair with food. I’m not going to say we do not. We definitely do. You know, I do not think we can de-program that. No, we cannot de-program that. You can tweak what we eat so that we can still eat what we used to and, you know, with less calories. But you cannot be telling me about loads of fish or soup or salads. I do not do salads unless I’ve got my main meal with the salad, so you cannot just tell me, have a salad on its own. So I need food with the salad. So that’s what we eat. It needs to be tweaked for what we eat because if I eat salad, I know if I eat salad with a little piece of meat, I’m still hungry. And if I’m still hungry, I’m going to want to still eat, whereas for another body, you know, another group of people, salads are fine. But no, for me, if I have that, I’m still going to eat and then I’ll be taking in too much. So that’s why I said the plans need to be tweaked to what we are. Normal eating patterns. Yeah. OK. Yeah” (Claudette, 50–54 yrs.).

“The only thing I can say is that the nutritional information on, regarding traditional foods is far less readily available than, you know, whatever, like your fish and chips or your Caesar salad and stuff like that. Yes, that’s the only thing I’ll mention I’d say on that really” (Oluseyi, 35–39 yrs.).

As previously noted, lack of engagement in commercial services where the individual has to pay out of pocket might partly relate to affordability. Service providers in private practices and from Black ethnic groups indicated that, among those with sufficient income, there was an active demand for their services, and that those clients also sought culturally tailored content ideally delivered by someone of similar identity. This provides further insight into the central importance and value of such tailoring,

“You know, healthy eating does not need to be Eurocentric. You can follow healthy eating advice and have a healthy life eating, you know, a traditional Afro Caribbean diet. I mean, yes, there are parts of it, which are indulgent. But does not every culture have that side to it? Why cannot people see the healthy side to it as well? So I’m a big advocate for that. And I think that’s probably where my success is and why people think oh, okay, let us, you know, talk it out. She might know what she’s talking about” (Sabryna, private sector dietitian).

##### Education and psychosocial support

Participants alluded to the need for general healthy living education. They also specifically mentioned the need to emphasise the importance of physical activity as part of that education. In addition, throughout the discussions and across the diversity of the sample the need for support and counselling to underpin services was conveyed. This included sensitivity around the psychological distress associated with obesity, but also support for stress and mental health concerns more generally,

“So it’s almost like I want to say if you do any weight loss approaches, you have to just understand how personal it is and how often sometimes it’s like when you are overweight, you might feel like a bit of a failure or like you cannot even control your weight, and if you cannot control your weight, how can you control your life? How do you control your destiny and your direction? So, I think it’s just be like, to take into account if you do any intervention like just think, like, and be gentle with people because everyone’s really different, and what you think they might be feeling they are probably not, it could just be something else and the best way to know is to ask them” (Emilie, 40–44 yrs.).

## Discussion

This novel qualitative study obtained views on weight management from UK women and men from Black ethnic groups living with excess weight, as well as from weight management service providers. The results highlighted a view of life that was positive but marred by racism and, for some public sector service providers from Black ethnic groups, their marginalised position prevented them giving the best support to clients of shared ethnicity. Weight gain was attributed by participants to unhealthy behaviours and the environment, and improving appearance and preventing ill health were key motivators for weight loss. Participants indicated that they relied on self-help to address their overweight, with general practice contested as a source of support for weight management. Anticipated or experienced racism accounted for some of the lack of engagement with services. Participants and service providers agreed on the lack of relevance of services, including limited culturally tailored resources. Community based, ethnically matched, and flexibly delivered weight management services for UK Black people was postulated as ideal. Overall, a number of recommendations for research and practice to improve weight management support for UK Black women and men can be extrapolated from our study results; either directly relayed by participants and service providers or intuited more broadly from the interpretation of the findings. These recommendations are summarised in Table 2.

**Table 2:** Recommendations for service development and research.

Recommendations
**Service development**
• Address cultural and social barriers to engagement in services, to include: • Investment in tailored self-help services such as digital applications; • Tailored dietary advice and recipe analysis; • Flexible timing and delivery platforms; • Mental health support; • Incentives to make take up of publicly and out of pocket funded services attractive to participants. • Enhance partnerships between communities, primary care, service providers, and researchers, bridged by community organisations, to embed services locally. • Utilise these partnerships to prioritise codesign approaches in service improvement and development of new programmes. • Incentivise service providers to take local race equality action, to include: • Diversifying the weight management workforce; • Increasing opportunities for ethnic matching between service users and practitioners. This could include training volunteers; • Providing training in anti-racism and cross-cultural competency
**Research**
• Increase support for research evaluating the effectiveness of race equality actions (as noted above) on improving weight management (and other health) outcomes. • Engage with stakeholder partnerships in codesigning, documenting, and evaluating new weight management programmes. • Improve the quality of ethnic group monitoring data in service and programme evaluations to enable localised understanding of inequalities and inform measures to address them. • Support equitable research partnerships between the UK and the home countries of Black ethnic migrants, facilitating bidirectional knowledge exchange on intervention development and implementation.

A range of reports, resources, and calls for research and action indicate increased recognition that ethnic inequality, and the role of racism and persistent exclusion in medicine and health, should be investigated (53–55). Prompted by these edicts, in the context of the inescapable spotlight on race inequalities cast by the COVID-19 pandemic, the NHS Race and Health Observatory^2^ was formed in 2021. The independent organisation brings issues of racism and health into the mainstream and is a vital opportunity for concerted action to address inequalities in access, experience, and outcomes (56) and highlights the scale of the work to be done to achieve meaningful change for Black and other underserved ethnic groups.

Marginalisation of Black people in the workplace has been identified in a range of UK professions and organisations including the NHS, for example (57). Our study confirmed lived experience of overt bias, inequality and discrimination. In addition it adds examples of what scholars refer to as “identity shifting” [(58), p. 154], among the service providers drawn from a range of occupations, from behavioural weight management services only to a wider range of specialist services. Defined as changing one’s behaviour in inter-cultural situations to become more socially invisible and counteract stereotyping and discrimination, identity shifting can be physically and emotionally costly (58). As we see in the current study (Theme 1, *Bringing your “big self” to work* subtheme), identity shifting could hamper the ability to fully support same-ethnicity clients. Due to a lack of proven national interventions, local action on equality, diversity, and inclusion is key (59).

The importance of traditional African or Caribbean food for these populations has consistently been noted across relevant (though limited) UK literature, regardless of whether the focus is on adults, children and families, or those with specific health conditions (60–62). Barriers to healthy eating as expressed by the participants, notably the cost of healthier food and easy access to fast food, are similarly well documented (22). The current study adds the nuanced detail of traditional eating being viewed as inconsistent with healthy eating, a view potentially shaped by advice from unqualified practitioners. Barriers to physical activity such as time and cost constraints are commonly noted in the literature; however the wider dimensions of disadvantage which are patterned by ethnicity (63) means that these generic barriers may be disproportionately experienced (64). Explicit removal of such barriers requires understanding of their underlying mechanisms [e.g., the impact on finances and workload of supporting families in home countries (65)]. Seen through a race equity lens, free availability of weight management programmes does address structural disadvantage on one hand, but as well as being affordable there is a need to consider the multiple and varied layers of individuals’ lived experiences to achieve equality of access (66), including opportunity costs as identified above.

Interaction with GPs and other primary healthcare professionals is a potentially important avenue to receiving weight management support, but was not commonly sought by participants. Prior experiences or anticipation of discrimination or inadequate care were cited as barriers to engagement. If engagement could be improved, even a short conversation with a healthcare professional can have a small impact on weight loss (67) and referral in these settings is a key route to accessing services free of charge. Guidance has been developed for health professionals to improve communication on weight with patients (68–70). Understanding of the subtleties of motivations, preferred language, and perspectives on overweight (such as it being a personal and not a clinical issue) could add insight to leverage points for effective conversations about weight with Black ethnic groups. For example, a positive view of a larger body size has been previously noted (19, 20) and a similarly positive view was partly the case in the current study. However, this was also bound up with participants wanting to lose weight for both appearance and health, or was ascribed to others but not themselves, further contributing to observations of a shift in perspectives (20).

More widely, participants were not aware of publicly funded weight management services, or their expectation or experience was that services did not suit their needs. Community based and cultural tailoring of programmes were consistently recommended by participants and service providers to address service limitations. The scaling up of community-centred approaches is embedded in UK policy to address health inequalities such as within Public Health England’s “family of approaches” (71) and NICE guidance (72). Methods employed by tailored private services to attract clientele, such as advertising around important dates for target groups and linking discounts with local food outlets, may translate to public sector services to improve uptake (73), and could be part of an effective communication strategy. Some participants drew on advice in online content from practitioners in home countries. There is some concern among public health nutrition scientists and policy makers around reliance on advice provided by unaccredited nutritionists due to lack of capacity in low resource countries (74). Development of weight management interventions for Black ethnic groups would benefit from equitable partnership between institutions in high and low income countries, where bidirectional learning could build capacity for implementing low-cost programmes in both settings (75).

The strong preference for ethnic concordance between service providers and clients is at odds with previous research which suggests more ambiguity (18). It is possible that ethnic-matching of researchers and participants drew out more honest accounts (76), as the detailed explanations for why concordance is important suggests that the opinions stated were not simply a different form of social desirability bias. There are no quick fixes to achieving the necessary workforce equality to meet this need (59). Therefore building cross-cultural competency into training of weight management service providers (77) is essential, and previously noted in relation to UK Black ethnic groups (25), and other minoritised groups in Europe (78, 79). This would be most valuable as part of the wider process of decolonising healthcare (80). Further development, evidence-based improvement, and dissemination of tailored resources that focus on traditional foods and their underpinning composition data is needed within the systemic changes outlined. This is labour intensive work for which the prioritisation of adequate funding is needed (81, 82). Holistic programmes which include flexible delivery and support for stress and mental health concerns, are also key elements of effective tailoring identified by participants, contemporaneously reported in underserved Black groups elsewhere (73).

A key strength of our study is its novel contribution to the existing evidence base. Few previous studies conducted in the UK have been identified with which to directly contrast our findings. Comparatively, we included men and women from the general population of Black ethnic groups living with excess weight, as opposed to a focus on subgroups with specific co-morbid health outcomes, such as living with T2D. Additionally, no other UK study has directly drawn on peoples’ experience of endemic discrimination to provide context to and direct links with weight management experiences. The study does have limitations. Although there is some socio-economic diversity in the sample, online/ remote recruitment methods did not work well for reaching populations of lower socio-economic circumstances. Whereas conducting interviews by phone was not problematic, the exchange of documents and completion of forms and questionnaires electronically is not easy for those in less affluent circumstances due to lack of access to computing hardware and/ or software. Documents were posted to four participants on request, but this was associated with no further response from those participants. We used the framework method to identify “fair dealing” [(83), p. 51] in representing perspectives and data saturation; however, it is possible that other views and experiences were not captured. The study is based on a small convenience sample, which places limits on generalisability of the findings. However, empirical representativeness is not the purpose of qualitative research and the objectives of the study were met. It is likely that the findings are theoretically transferable (84), and therefore resonate for Black ethnic groups across different UK settings.

## Conclusion

The findings of our qualitative analysis of potential service users’ and weight management service providers’ views indicate that weight management support for UK Black women and men needs to align with the wider social and cultural contexts that shape their everyday lives. Cultural tailoring of the delivery and content of services, and cultural competency training are needed. These actions are required within systemic changes, such as evaluated interventions to address discrimination, to facilitate better engagement in services. Future research recommendations include engaging with stakeholder partnerships in the codesign and evaluation of new weight management programmes. The study is based on a small convenience sample, and online recruitment methods may not be optimum for reaching populations of lower socio-economic circumstances. However it is likely that the findings reflect shared meaning regarding weight management needs for Black ethnic groups across different UK settings. Our exploratory insights therefore provide the basis for advancing further work and research to improve existing services to address the weight-related inequality faced by UK Black ethnic groups.

## Data availability statement

The original contributions presented in the study are included in the article/Supplementary material, further inquiries can be directed to the corresponding author.

## Ethics statement

The study involved humans and was approved by Research Ethics Committee, School of Health, Leeds Beckett University (Ref number: 85617). The study was conducted in accordance with the local legislation and institutional requirements. The participants provided their written informed consent to participate in this study.

## Author contributions

MJM, LE, EA, and MvD contributed to conception and design of the study. OO, MJM, and ES collected the data. MJM analysed the data with contributions from OO and TA. MJM wrote the first draft of the manuscript. All authors contributed to the article and approved the submitted version.

## Footnotes

^1^ https://heal-d.org/

^2^ https://www.nhsrho.org/

## References

[1] Gov.UK. (2020). Overweight adults. Available at: https://Www.Ethnicity-Facts-Figures.Service.Gov.Uk/Health/Diet-and-Exercise/Overweight-Adults/Latest (Accessed Nov 16, 2021).

[2] Sze S Pan D Nevill CR Gray LJ Martin CA Nazareth J. Ethnicity and clinical outcomes in Covid-19: a systematic review and meta-analysis. EClinicalMedicine. (2020) 29–30:100630. doi: 10.1016/j.eclinm.2020.100630 PMC7658622 33200120

[3] Public Health England. (2020). Excess Weight and Covid-19: Insights from new evidence. Available at: https://Www.Gov.Uk/Government/Publications/Excess-Weight-and-Covid-19-Insights-from-New-Evidence (Accessed Nov 16, 2021).

[4] Public Health England. (2020). Covid-19: Review of Disparities in Risks and Outcomes. Available at: https://Www.Gov.Uk/Government/Publications/Covid-19-Review-of-Disparities-in-Risks-and-Outcomes (Accessed Nov 16, 2021).

[5] Public Health England. (2020). Beyond the data: understanding the impact of Covid-19 on Bame groups. Available at: https://assets.PublishingServiceGovUk/Government/Uploads/System/Uploads/Attachment_Data/File/892376/Covid_Stakeholder_Engagement_Synthesis_Beyond_the_Data.Pdf (Accessed Nov 16, 2021).

[6] Townsend MJ Kyle TK Stanford FC. Outcomes of Covid-19: disparities in obesity and by ethnicity/race. Int J Obes. (2020) 44:1807–9. doi: 10.1038/s41366-020-0635-2, PMID: 32647359 PMC7347050

[7] World Health Organization. Obesity: preventing and managing the global epidemic: report of a who consultation. WHO Technical Report Series 894. Geneva: WHO (2000). 11234459

[8] Razieh C Zaccardi F Davies MJ Khunti K Yates T. Body mass index and risk of Covid-19 across ethnic groups: analysis of UK biobank study. Diabetes Obes Metab. (2020) 22:1953–4. doi: 10.1111/dom.14125, PMID: 32602268 PMC7362044

[9] Sattar N Ho FK Gill JM Ghouri N Gray SR Celis-Morales CA. BMI and future risk for Covid-19 infection and death across sex, age and ethnicity: preliminary findings from UK biobank. Diabetes Metab Syndr Clin Res Rev. (2020) 14:1149–51. doi: 10.1016/j.dsx.2020.06.060, PMID: 32668401 PMC7326434

[10] Zheng Y Ley SH Hu FB. Global aetiology and epidemiology of type 2 diabetes mellitus and its complications. Nat Rev Endocrinol. (2018) 14:88–98. doi: 10.1038/nrendo.2017.151 29219149

[11] Kyrou I Randeva HS Tsigos C Kaltsas G Weickert MO. (2018). Clinical Problems Caused by Obesity. Available at: https://Www.Ncbi.Nlm.Nih.Gov/Books/Nbk278973/ (Accessed Nov 11, 2023). South Dartmouth (MA): Endotext [Internet].

[12] Meeks KA Freitas-Da-Silva D Adeyemo A Beune EJ Modesti PA Stronks K. Disparities in type 2 diabetes prevalence among ethnic minority groups resident in Europe: a systematic review and meta-analysis. Intern Emerg Med. (2016) 11:327–40. doi: 10.1007/s11739-015-1302-9, PMID: 26370238

[13] Caleyachetty R Barber TM Mohammed NI Cappuccio FP Hardy R Mathur R. Ethnicity-specific Bmi cutoffs for obesity based on type 2 diabetes risk in England: a population-based cohort study. Lancet Diabetes Endocrinol. (2021) 9:419–26. doi: 10.1016/S2213-8587(21)00088-7, PMID: 33989535 PMC8208895

[14] Liu J Davidson E Bhopal R White M Johnson M Netto G. Adapting health promotion interventions to meet the needs of ethnic minority groups: mixed-methods evidence synthesis. Health Technol Assess. (2012) 16:1–469. doi: 10.3310/hta16440, PMID: 23158845 PMC4780992

[15] Leung G Stanner S. Diets of minority ethnic groups in the UK: influence on chronic disease risk and implications for prevention. Nutr Bull. (2011) 36:161–98. doi: 10.1111/j.1467-3010.2011.01889.x

[16] National Institute for Health and Care Excellence (NICE). (2014). Managing Overweight and Obesity in Adults – Lifestyle Weight Management Services. Nice public health guidance 53. Available at: http://Www.Nice.Org.Uk/Guidance/Ph53 (Accessed June 9, 2022).

[17] Goff LM Rivas C Moore A Beckley-Hoelscher N Reid F Harding S. Healthy eating and active lifestyles for diabetes (HEAL-D), a culturally tailored self-management education and support program for type 2 diabetes in black-British adults: a randomized controlled feasibility trial. BMJ Open Diabetes Res Care. (2021) 9:e002438. doi: 10.1136/bmjdrc-2021-002438, PMID: 34518159 PMC8438730

[18] Goff LM Moore AP Harding S Rivas C. Development of healthy eating and active lifestyles for diabetes, a culturally tailored diabetes self-management education and support programme for black-British adults: a participatory research approach. Diabet Med. (2021) 38:e14594. doi: 10.1111/dme.14594, PMID: 33961307

[19] Moore AP Rivas CA Stanton-Fay S Harding S Goff LM. Designing the healthy eating and active lifestyles for diabetes (HEAL-D) self-management and support Programme for UK African and Caribbean communities: a culturally tailored, complex intervention under-pinned by behaviour change theory. BMC Public Health. (2019) 19:1–14. doi: 10.1186/s12889-019-7411-z 31429735 PMC6702734

[20] Shoneye C Johnson F Steptoe A Wardle J. A qualitative analysis of black and white British women’s attitudes to weight and weight control. J Hum Nutr Diet. (2011) 24:536–42. doi: 10.1111/j.1365-277X.2011.01198.x, PMID: 21838745

[21] Er V Lane JA Martin RM Persad R Chinegwundoh F Njoku V. Barriers and facilitators to healthy lifestyle and acceptability of a dietary and physical activity intervention among African Caribbean prostate cancer survivors in the UK: a qualitative study. BMJ Open. (2017) 7:e017217. doi: 10.1136/bmjopen-2017-017217 PMC5652511 29038181

[22] Osei-Kwasi HA Nicolaou M Powell K Terragni L Maes L Stronks K. Systematic mapping review of the factors influencing dietary behaviour in ethnic minority groups living in Europe: a Dedipac study. Int J Behav Nutr Phys Act. (2016) 13:1–17. doi: 10.1186/s12966-016-0412-8 27465354 PMC4964011

[23] Patel N Ferrer HB Tyrer F Wray P Farooqi A Davies MJ. Barriers and facilitators to healthy lifestyle changes in minority ethnic populations in the UK: a narrative review. J Racial Ethn Health Disparities. (2017) 4:1107–19. doi: 10.1007/s40615-016-0316-y, PMID: 27928772 PMC5705764

[24] Such E Salway S Copeland R Haake S Domone S Mann S. A formative review of physical activity interventions for minority ethnic populations in England. J Public Health. (2017) 39:e265–74. doi: 10.1093/pubmed/fdw126, PMID: 27899479 PMC6372170

[25] Goff LM Moore A Harding S Rivas C. Providing culturally sensitive diabetes self-management education and support for black African and Caribbean communities: a qualitative exploration of the challenges experienced by healthcare practitioners in inner London. BMJ Open Diabetes Res Care. (2020) 8:e001818. doi: 10.1136/bmjdrc-2020-001818, PMID: 33293296 PMC7725076

[26] Chouhan K Nazroo J. Health inequalities. Ethnicity, race and inequality in the UK-state of the nation. Bristol, UK: Policy Press (2020).

[27] Nazroo J. Tackling racism: moving beyond rhetoric to turn theory into practice. BMJ. (2022) 378:o1597. doi: 10.1136/bmj.o1597 35938647

[28] Salway S Holman D Lee C McGowan V Ben-Shlomo Y Saxena S. Transforming the health system for the UK’S multiethnic population. BMJ. (2020) 368:m268. doi: 10.1136/bmj.m268 32047065

[29] Cerdeña JP Plaisime MV Tsai J. From race-based to race-conscious medicine: how anti-racist uprisings call us to act. Lancet. (2020) 396:1125–8. doi: 10.1016/S0140-6736(20)32076-6, PMID: 33038972 PMC7544456

[30] Leitch S Corbin JH Boston-Fisher N Ayele C Delobelle P Gwanzura Ottemöller F. Black lives matter in health promotion: moving from unspoken to outspoken. Health Promot Int. (2021) 36:1160–9. doi: 10.1093/heapro/daaa121, PMID: 33305322 PMC7953963

[31] Ruger J. Health and social justice. Lancet. (2004) 364:1075–80. doi: 10.1016/S0140-6736(04)17064-5, PMID: 15380968 PMC4006198

[32] Ormston R Spencer L Barnard M Snape D. The foundations of qualitative research In: Ritchie J Lewis J Nicholls CM Ormston R, editors. Qualitative research practice: a guide for social science students and researchers. London: SAGE Publications Ltd (2014). 1–26.

[33] Greenhalgh T Hinton L Finlay T Macfarlane A Fahy N Clyde B. Frameworks for supporting patient and public involvement in research: systematic review and co-design pilot. Health Expect. (2019) 22:785–801. doi: 10.1111/hex.12888, PMID: 31012259 PMC6737756

[34] Koh HK Oppenheimer SC Massin-Short SB Emmons KM Geller AC Viswanath K. Translating research evidence into practice to reduce health disparities: a social determinants approach. Am J Public Health. (2010) 100:S72–80. doi: 10.2105/AJPH.2009.167353, PMID: 20147686 PMC2837437

[35] Alderson P. Critical realism for health and illness research: a practical introduction. Bristol: Policy Press (2021).

[36] Tong A Sainsbury P Craig J. Consolidated criteria for reporting qualitative research (Coreq): a 32-item checklist for interviews and focus groups. Int J Qual Health Care. (2007) 19:349–57. doi: 10.1093/intqhc/mzm042, PMID: 17872937

[37] NICE. (2013). BMI: Preventing ill health and premature death in black, Asian and other minority ethnic groups. Public Health Guideline [Ph46]. Available at: https://Www.Nice.Org.Uk/Guidance/Ph46 (Accessed Nov 16, 2021). NICE London.

[38] Morse JM Barrett M Mayan M Olson K Spiers J. Verification strategies for establishing reliability and validity in qualitative research. Int J Qual Methods. (2002) 1:13–22. doi: 10.1177/160940690200100202

[39] Guest G Bunce A Johnson L. How many interviews are enough? An experiment with data saturation and variability. Field Methods. (2006) 18:59–82. doi: 10.1177/1525822X05279903

[40] Kang C Tomkow L Farrington R. Access to primary health care for Asylum seekers and refugees: a qualitative study of service user experiences in the UK. Br J Gen Pract. (2019) 69:e537–45. doi: 10.3399/bjgp19X701309, PMID: 30745354 PMC6617541

[41] Krieger N Smith K Naishadham D Hartman C Barbeau EM. Experiences of discrimination: validity and reliability of a self-report measure for population Health Research on racism and health. Soc Sci Med. (2005) 61:1576–96. doi: 10.1016/j.socscimed.2005.03.006 16005789

[42] Lewis JA Williams MG Peppers EJ Gadson CA. Applying intersectionality to explore the relations between gendered racism and health among black women. J Couns Psychol. (2017) 64:475–86. doi: 10.1037/cou0000231, PMID: 29048194

[43] Shelton RC Adsul P Oh A. Recommendations for addressing structural racism in implementation science: a call to the field. Ethn Dis. (2021) 31:357–64. doi: 10.18865/ed.31.S1.357, PMID: 34045837 PMC8143847

[44] Wagner JA Osborn CY Mendenhall EA Budris LM Belay S Tennen HA. Beliefs about racism and health among African American women with diabetes: a qualitative study. J Natl Med Assoc. (2011) 103:224–33. doi: 10.1016/S0027-9684(15)30298-4, PMID: 21528110 PMC3082367

[45] Coupe N Cotterill S Peters S. Tailoring lifestyle interventions to low socio-economic populations: a qualitative study. BMC Public Health. (2018) 18:1–15. doi: 10.1186/s12889-018-5877-8 PMC6076398 30075716

[46] Rozenblum R De La Cruz BA Nolido NV Adighibe I Secinaro K McManus KD. Primary care patients’ and providers’ perspectives about an online weight management program: a qualitative study. J Gen Intern Med. (2019) 34:1503–21. doi: 10.1007/s11606-019-05022-6, PMID: 31152361 PMC6667547

[47] Stuber JM Middel CN Mackenbach JD Beulens JW Lakerveld J. Successfully recruiting adults with a low socioeconomic position into community-based lifestyle programs: a qualitative study on expert opinions. Int J Environ Res Public Health. (2020) 17:2764. doi: 10.3390/ijerph17082764, PMID: 32316344 PMC7215437

[48] Arthur A Mitchell M Lewis J McNaughton NC. Designing fieldwork In: Ritchie J Lewis J McNaughton Nicholls C Ormston R, editors. Qualitative research practice: a guide for social science students and researchers. 2nd ed. London: SAGE (2014). 147–76.

[49] Braun V Clarke V. Thematic analysis: a practical guide. London: Sage (2021).

[50] Braun V Clarke V. Using thematic analysis in psychology. Qual Res Psychol. (2006) 3:77–101. doi: 10.1191/1478088706qp063oa

[51] Gale NK Heath G Cameron E Rashid S Redwood S. Using the framework method for the analysis of qualitative data in multi-disciplinary Health Research. BMC Med Res Methodol. (2013) 13:1–8. doi: 10.1186/1471-2288-13-117 24047204 PMC3848812

[52] Steim RI Nemeroff CJ. Moral overtones of food: judgments of others based on what they eat. Personal Soc Psychol Bull. (1995) 21:480–90. doi: 10.1177/0146167295215006

[53] Chew M Das P Aujla M Horton R. Advancing racial and ethnic equity in science, medicine, and health: a call for papers. Lancet. (2021) 398:1287–9. doi: 10.1016/S0140-6736(21)02095-X 34592136

[54] Kmietowicz Z. (Ed). (2020). Racism in medicine [Special Issue]. BMJ; 368. Available at: https://Www.Bmj.Com/Racism-in-Medicine (Accessed Nov 16, 2021).

[55] Public Health England. (2018). Local action on health inequalities: understanding and reducing ethnic inequalities in health. Available at: https://assets.Publishing.Service.Gov.Uk/Government/Uploads/System/Uploads/Attachment_Data/File/730917/Local_Action_on_Health_Inequalities.Pdf (Accessed Nov 16, 2021).

[56] Iacobucci G. NHS race and health observatory appoints global experts on inequalities to its board. BMJ. (2021):n122. doi: 10.1136/bmj.n122 33446484

[57] NHS England. (2021). Medical workforce race equality standard (MWRES): WRES indicators for the medical workforce 2020. Available at: https://Www.England.Nhs.Uk/Wp-Content/Uploads/2021/07/Mwres-Digital-2020_Final.Pdf (Accessed Nov 16, 2021).

[58] Dickens DD Womack VY Dimes T. Managing hypervisibility: an exploration of theory and research on identity shifting strategies in the workplace among black women. J Vocat Behav. (2019) 113:153–63. doi: 10.1016/j.jvb.2018.10.008

[59] Ross S Jabbal J Chauhan K Maguire D Randhawa M Dahir S. Workforce race inequalities and inclusion in NHS providers. London: King’s Fund (2020).

[60] Maynard M Baker G Harding S. Exploring childhood obesity prevention among diverse ethnic groups in schools and places of worship: recruitment, acceptability and feasibility of data collection and intervention components. Prev Med Rep. (2017) 6:130–6. doi: 10.1016/j.pmedr.2017.02.019, PMID: 28316908 PMC5345967

[61] Ochieng L Amaugo L Ochieng BM. Developing healthy weight maintenance through co-creation: a partnership with black African migrant community in East Midlands. Eur J Pub Health. (2021) 31:487–93. doi: 10.1093/eurpub/ckaa222 33532825

[62] Rawlins E Baker G Maynard M Harding S. Perceptions of healthy eating and physical activity in an ethnically diverse sample of young children and their parents: the deal prevention of obesity study. J Hum Nutr Diet. (2013) 26:132–44. doi: 10.1111/j.1365-277X.2012.01280.x, PMID: 22827466 PMC3618369

[63] Centre for Social Justice. (2020). Facing the Facts: Ethnicity and Disadvantage in Britain. Disparities in Education, Work, and Family. Available at: https://Www.Centreforsocialjustice.Org.Uk/Wp-Content/Uploads/2020/11/Csjj8513-Ethnicity-Poverty-Report-Final.Pdf (Accessed June 15, 22).

[64] Koshoedo SA Paul-Ebhohimhen VA Jepson RG Watson MC. Understanding the complex interplay of barriers to physical activity amongst black and minority ethnic groups in the United Kingdom: a qualitative synthesis using meta-ethnography. BMC Public Health. (2015) 15:1–16. doi: 10.1186/s12889-015-1893-0 26164652 PMC4499183

[65] Silva CV Klimaviciute L. (2020). Migrant remittances to and from the UK. Available at: https://Migrationobservatory.Ox.Ac.Uk/Wp-Content/Uploads/2016/04/Briefing-Migrant-Remittances-to-and-from-the-Uk.Pdf (Accessed Nov 16, 2021).

[66] Netto G Bhopal R Lederle N Khatoon J Jackson A. How can health promotion interventions be adapted for minority ethnic communities? Five principles for guiding the development of behavioural interventions. Health Promot Int. (2010) 25:248–57. doi: 10.1093/heapro/daq012, PMID: 20299500

[67] Aveyard P Lewis A Tearne S Hood K Christian-Brown A Adab P. Screening and brief intervention for obesity in primary care: a parallel, two-arm, randomised trial. Lancet. (2016) 388:2492–500. doi: 10.1016/S0140-6736(16)31893-1, PMID: 27789061 PMC5121130

[68] Albury C Strain WD Le Brocq S Logue J Lloyd C Tahrani A. The importance of language in engagement between health-care professionals and people living with obesity: a joint consensus statement. Lancet Diabetes Endocrinol. (2020) 8:447–55. doi: 10.1016/S2213-8587(20)30102-9 32333880

[69] OHID. (2021). Reference guide for primary care networks and staff on the healthy weight coach Elearning Programme. Available at: https://Www.Gov.Uk/Government/Publications/Healthy-Weight-Coach-Elearning-Programme-for-Primary-Care-Networks-Healthcare-Practices-and-Pharmacies (Accessed June 15, 2022).

[70] Thompson L Aveyard P Jebb S Blackshaw J Coulton V. (2017). Let’s talk about weight: a step-by-step guide to brief interventions with adults for health and care professionals. Available at: https://assets.Publishing.Service.Gov.Uk/Government/Uploads/System/Uploads/Attachment_Data/File/737903/Weight_Management_Toolkit_Let_S_Talk_About_Weight.Pdf (Accessed Nov 16, 2021).

[71] Public Health England. (2015). A guide to community-centred approaches for health and wellbeing. Available at: https://assets.Publishing.Service.Gov.Uk/Government/Uploads/System/Uploads/Attachment_Data/File/768979/a_Guide_to_Community-Centred_Approaches_for_Health_and_Wellbeing__Full_Report_.Pdf (Accessed Nov 16, 2021).

[72] NICE. (2016). Community Engagement: Improving Health and Wellbeing and Reducing Health Inequalities. Available at: https://Www.Nice.Org.Uk/Guidance/Ng44/Resources/Community-Engagement-Improving-Health-and-Wellbeing-and-Reducing-Health-Inequalities-Pdf-1837452829381. (Accessed Nov 16, 2021).

[73] James DC Harville C II McQueen DS Facey JA. I want a program that looks at my whole life: a focus group study on the ideal components for an Mhealth weight management program for African American women. J Acad Nutr Diet. (2022) 122:139–48. doi: 10.1016/j.jand.2021.06.310 34351276

[74] Delisle H Shrimpton R Blaney S Du Plessis L Atwood S Sanders D. Capacity-building for a strong public health nutrition workforce in low-resource countries. Bull World Health Organ. (2017) 95:385–8. doi: 10.2471/BLT.16.174912, PMID: 28479641 PMC5418830

[75] Oni T Assah F Erzse A Foley L Govia I Hofman KJ. The global diet and activity research (Gdar) network: a global public health partnership to address upstream Ncd risk factors in urban low and middle-income contexts. Glob Health. (2020) 16:1–11. doi: 10.1186/s12992-020-00630-y PMC7570103 33076935

[76] Ochieng BM. “You know what I mean:” the ethical and methodological dilemmas and challenges for black researchers interviewing black families. Qual Health Res. (2010) 20:1725–35. doi: 10.1177/1049732310381085, PMID: 20729502

[77] Handtke O Schilgen B Mösko M. Culturally competent healthcare–a scoping review of strategies implemented in healthcare organizations and a model of culturally competent healthcare provision. PLoS One. (2019) 14:e0219971. doi: 10.1371/journal.pone.0219971, PMID: 31361783 PMC6667133

[78] Admiraal WM Vlaar EM Nierkens V Holleman F Middelkoop BJC Stronks K. Intensive lifestyle intervention in general practice to prevent type 2 diabetes among 18 to 60-year-old south Asians: 1-year effects on the weight status and metabolic profile of participants in a randomized controlled trial. PLoS One. (2013) 8:e68605. doi: 10.1371/journal.pone.0068605 23894322 PMC3718785

[79] Nicolaou M Vlaar E Van Valkengoed I Middelkoop B Stronks K Nierkens V. Development of a diabetes prevention program for Surinamese south Asians in the Netherlands. Health Promot Int. (2014) 29:680–91. doi: 10.1093/heapro/dat018 23564419

[80] Mullard JCR. Race, racism and anthropology: decolonising health inequality in a time of Covid-19. Med Anthropol Theory. (2021) 8:1–19. doi: 10.17157/mat.8.1.5112

[81] Apekey T Copeman J Kime N Tashani O Kittana M Walsh D. Methods of producing new nutrient data for popularly consumed multi ethnic foods in the UK. J Food Compos Anal. (2019) 78:9–18. doi: 10.1016/j.jfca.2019.01.011

[82] Apekey TA Copeman J Kime NH Tashani OA Kittaneh M Walsh D. Nutrient composition of popularly consumed African and Caribbean foods in the UK. Foods. (2019) 8:500. doi: 10.3390/foods8100500, PMID: 31618872 PMC6835955

[83] Mays N Pope C. Assessing quality in qualitative research. BMJ. (2000) 320:50–2. doi: 10.1136/bmj.320.7226.50, PMID: 10617534 PMC1117321

[84] Lewis J Ritchie J Ormston R Morrell G. Generalising from qualitative research In: Ritchie J Lewis J McNaughton Nicholls C Ormston R, editors. Qualitative research practice: a guide for social science students and researchers. 2nd ed. London: SAGE (2014). 347–66.

