# Supplementary material for: Improving adult behavioural weight management services for diverse UK Black Caribbean and Black African ethnic groups: a qualitative study of insights from potential service users and service providers
Source: Front Public Health. 2023 Nov 23;11:1239668. doi: 10.3389/fpubh.2023.1239668 (PMC10701265; doi:10.3389/fpubh.2023.1239668)
Supplement: Supplementary file 1 [file Table_1.docx]

**Supplementary File 1. Consolidated criteria for reporting qualitative studies (COREQ): 32-item checklist [21]**

| **Item** | **Guide questions/description** | **Reported on Page #** |
| --- | --- | --- |
| **Domain 1: Research team and reﬂexivity** |  |  |
| ***Personal Characteristics*** | | |
| 1. Interviewer/facilitator | Which author/s conducted the interviews or focus groups? | 4 |
| 2. Credentials;  3. Occupation;  4. Gender;  5. Experience and training | What were the researcher’s credentials? E.g. PhD, MD  What was their occupation at the time of the study?  Were the researchers male or female?  What experience or training did the researchers have? | 3 |
| ***Relationship with participants*** | | |
| 6. Relationship established;  7. Participant knowledge of the interviewer | Was a relationship established prior to study commencement?  What did the participants know about the researcher? e.g. personal goals, reasons for doing the research | 3 |
| 8. Interviewer characteristics | What characteristics were reported about the interviewer/facilitator? e.g. Bias, assumptions, reasons and interests in the research topic | As reported for items 1-5 |

| **Domain 2: study design** |  |  |
| --- | --- | --- |
| ***Theoretical framework*** | | |
| 9. Methodological orientation and Theory | What methodological orientation was stated to underpin the study? e.g. grounded theory, discourse analysis, ethnography, phenomenology, content analysis | 3  4 |
| ***Participant selection*** | | |
| 10. Sampling | How were participants selected? e.g. purposive, convenience, consecutive, snowball | 3-4 |
| 11. Method of approach | How were participants approached? e.g. face-to-face, telephone, mail, email | 3 |
| 12. Sample size | How many participants were in the study? | 5  (Table 1) |
| 13. Non-participation | How many people refused to participate or dropped out? Reasons? | Not known |
| ***Setting*** | | |
| 14. Setting of data collection | Where was the data collected? e.g. home, clinic, workplace | 3 |
| 15. Presence of non-participants | Was anyone else present besides the participants and researchers? | No |
| 16. Description of sample | What are the important characteristics of the sample? e.g. demographic data, date | 4 (Table 1) |
| ***Data collection*** | | |
| 17. Interview guide | Were questions, prompts, guides provided by the authors?  Was it pilot tested? | Supplementary file 2  4 |
| 18. Repeat interviews | Were repeat inter views carried out? If yes, how many? | No |
| 19. Audio/visual recording  20. Field notes  21. Duration | Did the research use audio or visual recording to collect the data?  Were ﬁeld notes made during and/ or after the interview or focus group?  What was the duration of the interviews or focus groups? | 4 |
| 22. Data saturation | Was data saturation discussed? | 4 |
| 23. Transcripts returned | Were transcripts returned to participants for comment and/or correction? | No |
| **Domain 3: analysis and ﬁndings** |  |  |
| ***Data analysis*** | | |
| 24. Number of data coders | How many data coders coded the data? | Two |
| 25. Description of the coding tree | Did authors provide a description of the coding tree? | Supplementary File 3 |
| 26. Derivation of themes | Were themes identiﬁed in advance or derived from the data? | 4 |
| 27. Software | What software, if applicable, was used to manage the data? | N/A |
| 28. Participant checking | Did participants provide feedback on the ﬁndings? | No |
| ***Reporting*** | | |
| 29. Quotations presented | Were participant quotations presented to illustrate the themes/ﬁndings? Was each quotation identiﬁed? e.g. participant number | 5-12 |
| 30. Data and ﬁndings consistent | Was there consistency between the data presented and the ﬁndings? | 5-12 |
| 31. Clarity of major themes | Were major themes clearly presented in the ﬁndings? | 5-12 |
| 32. Clarity of minor themes | Is there a description of diverse cases or discussion of minor themes? | 6-12 |
