# Supplementary material for: Improving adult behavioural weight management services for diverse UK Black Caribbean and Black African ethnic groups: a qualitative study of insights from potential service users and service providers
Source: Front Public Health. 2023 Nov 23;11:1239668. doi: 10.3389/fpubh.2023.1239668 (PMC10701265; doi:10.3389/fpubh.2023.1239668)
Supplement: Supplementary file 2 [file Table_2.DOCX]

**Supplementary File 2. Interview topics and prompts**

| **A. Potential service users** |
| --- |
| 1. **Introduction**  - Introduction to researcher - Consent to participate and to record the session - Discussion of the participant information sheet - Outline of the structure of interview |
| 1. **Life history and migration narratives:**  - Place of birth and growing up - [If born abroad] life prior to coming to the UK - Reasons for coming to the UK - Life here in the UK |
| 1. **Racism and other discrimination**  - Experiences of racism and other forms of discrimination - Being treated unfairly because of your ethnicity, race, colour or religion. - Worry or stress due to being treated unfairly because of ethnicity, race, colour or religion. - Recommendations for addressing racism/discrimination in health related settings. |
| 1. **Constructions of health/ healthy weight**  - Views on own health - Views on excess body weight:   *Causes*  *Risk factors*  *Impact on quality of life and mental health*  *Weight management options*  *Meaning of BMI*   - Weight across life course - Any struggles with weight |
| 1. **Exploring associations between life stories, health and weight**  - Events in life and health & wellbeing - (if born abroad) Migration journeys and health & wellbeing - … regarding weight in particular |
| 1. **Motivation**  - Reasons for wanting to lose weight - When first wanted to lose weight - Relationship with food - Level of interest in physical activity/ exercise - Advice/ or support received regarding PA/ exercise |
| 1. **Access to services**  - Awareness of weight management services or support   *Local authority initiatives*  *Community-based third sector organisations*  *Commercial organisations e.g. slimming world etc*  *Bariatric surgery/ medication*   - Own approach to weight management - Any plans to approach managing your weight?   *Asking for help*  *Self-help*  *Family friends/ word of mouth*  *Internet/social media – official and unofficial*  *GP/health professional*  *Community-based services*   - What prompted approach to weight loss |
| 1. **Barriers and facilitators**  - Challenges faced on weight loss journey   *Lack of knowledge on healthy eating with regards to traditional foods*  *Lack of service provider/ health professional knowledge on traditional foods*  *Lack of support*  *Financial issues*  *Health problem*  *Social life*   - Encouragement on the journey   *Health*  *Appearance*  *Family and friends*  *Access to services*  *community/ community leaders*   - Household members involved in food shopping and preparation - Views on weight management services and support for people from a similar background   *Knowledge of working with ethnic/ cultural groups which are different from their own [Cultural competence of providers]*  *Culturally appropriateness of foods and exercise recommended/discussed*  *Access*   - Importance of health professionals from the same ethnic group - Importance of health professionals of the same gender - Importance of health professionals of the same body shape and size - What an ideal weight management service would look like   *Specific content*  *Remote/ digital or face to face* |
| 1. **Conclusion**  - Summary of the discussion - Any final comments |

| **B. Service providers** |
| --- |
| 1. **Introduction:**   As above |
| 1. **Weight management programme and role**  - Nature of the weight management programme involved in providing - Any inclusion of resources for participants on the programme - The decision making process on the approach to weight management with individuals - Description of the specific role played in the programme   *Design*  *Delivery*  *Modification*  *Lead*  *Multi-disciplinary team*   - How the programme/s is delivered - Where delivered [location if face to face] - Programme/s components |
| 1. **Programme participants**  - How are users recruited/ referred to the programme?   *Varied recruitment strategies*  *Methods of awareness raising of the programme*  *Targets (numbers and characteristics of users)*  *Planned vs achieved numbers*   - Any (un)expected challenges during recruitment - Strategies to address the (un)expected challenges - Most successful approach - convenience of this approach - Least successful approach - convenience is this approach - The main ethnic groups among the programme participants - … specifically Black African and Black Caribbean men and women - Reasons for being successful/ unsuccessful in recruiting participants from these ethnic groups and/or diverse groups more widely   *Incentives to retain participants from diverse ethnic/ social groups*   - Extent programme/s tailored for different ethnic groups - Process of cultural adaptation / why there is little cultural adaptation - Confidence in working cross culturally   *Own ethnicity/ language ability*  *Specific training in cultural competency/ unconscious bias/ race equality* |
| 1. **Effectiveness, barriers and facilitators**  - Effectiveness of the programme/s - Measure/s effectiveness - For whom the programme is successful - Main facilitators that support weight management in the programme/s - ...and specifically for Black African and Black Caribbean ethnic groups - Main barriers to supporting weight management in the programme/s - ...and specifically for Black African and Black Caribbean ethnic groups   *Referral/ recruitment*  *Norms/ beliefs*  *Socio-economic factors*  *Environmental factors*  *Racism/ discrimination*   - What would help address the barriers - Any additional resources/ intervention components useful for the programme users - Why these are not already provided - Recommendations for future programmes targeting Black ethnic groups [what an excellent programme would look like] |
| 1. **Conclusion**   As above |
