# Supplementary material for: Improving adult behavioural weight management services for diverse UK Black Caribbean and Black African ethnic groups: a qualitative study of insights from potential service users and service providers
Source: Front Public Health. 2023 Nov 23;11:1239668. doi: 10.3389/fpubh.2023.1239668 (PMC10701265; doi:10.3389/fpubh.2023.1239668)
Supplement: Supplementary file 3 [file Presentation_1.PPTX]

## Slide 1
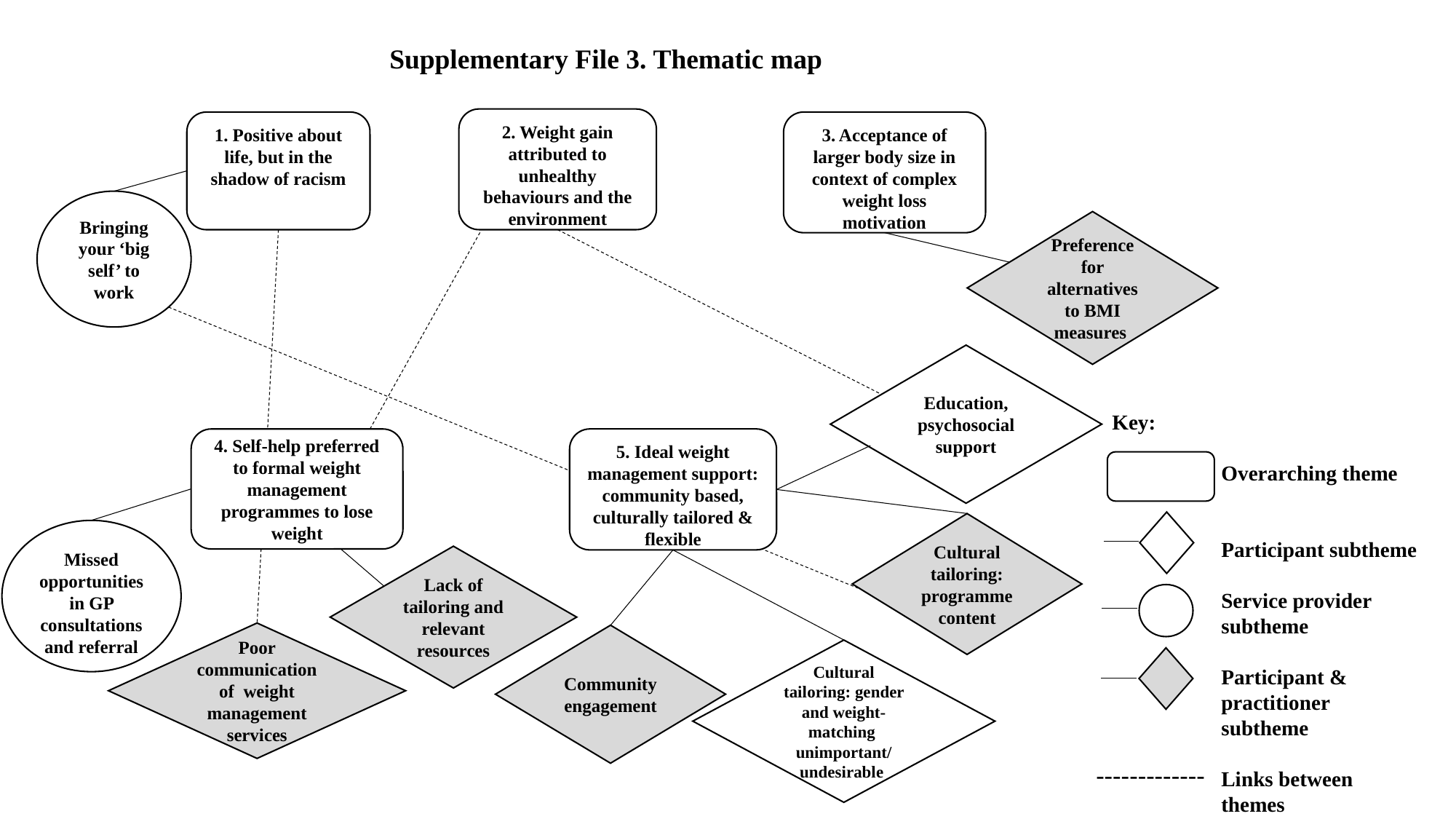

Supplementary File 3. Thematic map
2. Weight gain attributed to unhealthy behaviours and the environment
1. Positive about life, but in the shadow of racism
3. Acceptance of larger body size in context of complex weight loss motivation
Bringing your ‘big self’ to work
Preference for alternatives to BMI measures
Education, psychosocial support
5. Ideal weight management support: community based, culturally tailored & flexible
Cultural tailoring: programme content
Missed opportunities in GP consultations and referral
Lack of tailoring and relevant resources
Community engagement
Cultural tailoring: gender and weight-matching unimportant/ undesirable
4. Self-help preferred to formal weight management programmes to lose weight
Poor communication of weight management services
Key:
	Overarching theme
	Participant subtheme
	Service provider 	subtheme
	Participant & 	practitioner
	subtheme
	Links between 	themes
0
-------------
